# Development of dopant-free conductive bioelastomers

**DOI:** 10.1038/srep34451

**Published:** 2016-09-30

**Authors:** Cancan Xu, Yihui Huang, Gerardo Yepez, Zi Wei, Fuqiang Liu, Alejandro Bugarin, Liping Tang, Yi Hong

**Affiliations:** 1 Department of Bioengineering, University of Texas at Arlington, Arlington, TX 76019, USA; 2 Joint Biomedical Engineering Program, University of Texas Southwestern Medical Center, Dallas, TX 75093, USA; 3 Department of Chemistry and Biochemistry, University of Texas at Arlington, Arlington, Texas 76019, USA; 4 Department of Material Science and Engineering, University of Texas at Arlington, Arlington, TX 76019, USA

## Abstract

Conductive biodegradable materials are of great interest for various biomedical applications, such as tissue repair and bioelectronics. They generally consist of multiple components, including biodegradable polymer/non-degradable conductive polymer/dopant, biodegradable conductive polymer/dopant or biodegradable polymer/non-degradable inorganic additives. The dopants or additives induce material instability that can be complex and possibly toxic. Material softness and elasticity are also highly expected for soft tissue repair and soft electronics. To address these concerns, we designed a unicomponent dopant-free conductive polyurethane elastomer (DCPU) by chemically linking biodegradable segments, conductive segments, and dopant molecules into one polymer chain. The DCPU films which had robust mechanical properties with high elasticity and conductivity can be degraded enzymatically and by hydrolysis. It exhibited great electrical stability in physiological environment with charge. Mouse 3T3 fibroblasts survived and proliferated on these films exhibiting good cytocompatibility. Polymer degradation products were non-toxic. DCPU could also be processed into a porous scaffold and in an *in vivo* subcutaneous implantation model, exhibited good tissue compatibility with extensive cell infiltration over 2 weeks. Such biodegradable DCPU with good flexibility and elasticity, processability, and electrical stability may find broad applications for tissue repair and soft/stretchable/wearable bioelectronics.

Conductive biomaterials, including conductive composites and polymers, have been explored for their application as smart scaffolds for tissue repair and regeneration^1^. It is believed that conductive biomaterials can deliver electrical stimulation to cells and modulate cell behavior (e.g., adhesion, migration, proliferation and differentiation)^1^,^2^. They can also promote repair and regeneration of various tissues, such as myocardium, nerve, muscle, skin and bone, compared with conventional insulate biodegradable polymers^3^,^4^,^5^,^6^. For example, aligned electrospun poly(ε-caprolactone)/polyaniline fibrous scaffolds could guide myoblast orientation and promote myotube formation^3^. Alginate scaffold incorporated with gold nanowires enhanced electromechanical coupling and contractile properties of cardiac patches, and promoted the growth and maturation of cardiac cells^4^. A multiwalled carbon nanotube (MWCNTs)-coated 3D collagen sponge was implanted in rat femur and significantly more bone formation was observed around the MWCNT-coated sponge than the uncoated sponge at 28 and 56 days^5^. Inner walls of nerve guidance channels coated with polypyrrole-based copolymers promoted neurite growth in the sciatic nerves of rats within 8 weeks^6^. Besides tissue engineering application, conductive biomaterials have great potential in other biomedical applications, such as electrically-triggered drug release, biosensors, and bioactuators^7^,^8^,^9^,^10^.

Current conductive composites contain biodegradable polymers (e.g., polylactide, polycaprolactone, and polyurethane) and organic conductive polymers (e.g., polyaniline, polypyrrole, and poly(3,4-ethylenedioxythiophene) (PEDOT)) or inorganic additives (e.g., carbon nanotubes, graphene, gold nanowire), in which the biodegradable polymers provide mechanical behavior and the non-degradable additives provide electric conductivity^4^,^11^,^12^,^13^. Biodegradable conductive polymers are synthesized through conjugating conductive segments with biodegradable polymers, such as polylactide-aniline pentamer-polylactide (PLA-AP-PLA)^2^, polypyrrole-co-polycaprolactone (PPy-PCL)^14^, and hyperbranched ductile polylactide (HPLA)-co-aniline tetramer (AT) (HPLAAT)^15^, which require mixing with dopants for conductivity. However, there are some major concerns about the use of these conductive biomaterials for tissue repair and regeneration. A dopant is required for conventional conductive polymers in the composites and the biodegradable conductive polymers to achieve high conductivity^16^,^17^. They are used to dope the polymer via physical mixture, and they can leach with time or electrical stimulus. This behavior not only deteriorates the electrical properties (e.g., conductivity and electrical stability) of conductive polymers but also their cytotoxicity^1^,^17^,^18^. Furthermore, the addition of dopants could influence other material properties of the conductive polymers^17^. For example, their mixture can increase the stiffness of conductive material, and negatively affect the material flexibility and elasticity, which could limit their application in soft tissue repair and regeneration and in soft/stretchable electronics^16^,^19^,^20^,^21^. An alternative approach to avoiding the dopant mixture is to covalently bind the dopants into a polymer constituent and then dope the conductive polymers^22^,^23^. A hybrid conductive hydrogel without the need for mobile doping ions was prepared with PEDOT evenly distributed in poly(vinyl alcohol) (PVA)/heparin methacrylate (Hep-MA) hydrogel. Heparin was covalently bound to the PVA backbone and doped the PEDOT^22^. The conductive hydrogel exhibited superior mechanical stability and retained superior electroactivity compared to metal electrodes. Unfortunately, the lack of biodegradability could limit the use of these hybrids in tissue engineering application. To overcome these drawbacks, we have developed a biodegradable conductive polymer with desirable electrical (stable electrochemical performance) and mechanical (robust, soft and elastic) properties without dopant mixture.

In this study, we have designed a dopant-free conductive elastomer by chemically linking biodegradable segments, conductive segments, and dopant molecules into one polymer chain. Specifically, a biodegradable polycaprolactone diol (PCL), conductive aniline trimer, and dopant dimethylolpropionic acid (DMPA) were linked into a polyurethane chain through hexadiisocyanate. The electrical, mechanical and biodegradable properties of the conductive polyurethane films were characterized. The electrical stability was evaluated under physiological conditions. Cytotoxicity of the conductive polyurethane degradation products and the cytocompatibility of the conductive films were assessed using mouse 3T3 fibroblasts. Furthermore, the dopant-free conductive polyurethane was processed into porous scaffolds using salt-leaching, and then implanted into a mouse subcutaneous model for *in vivo* biocompatibility evaluation.

## Results and Discussion

### Characterization of the dopant-free conductive polyurethane (DCPU)

**Figure 1:**
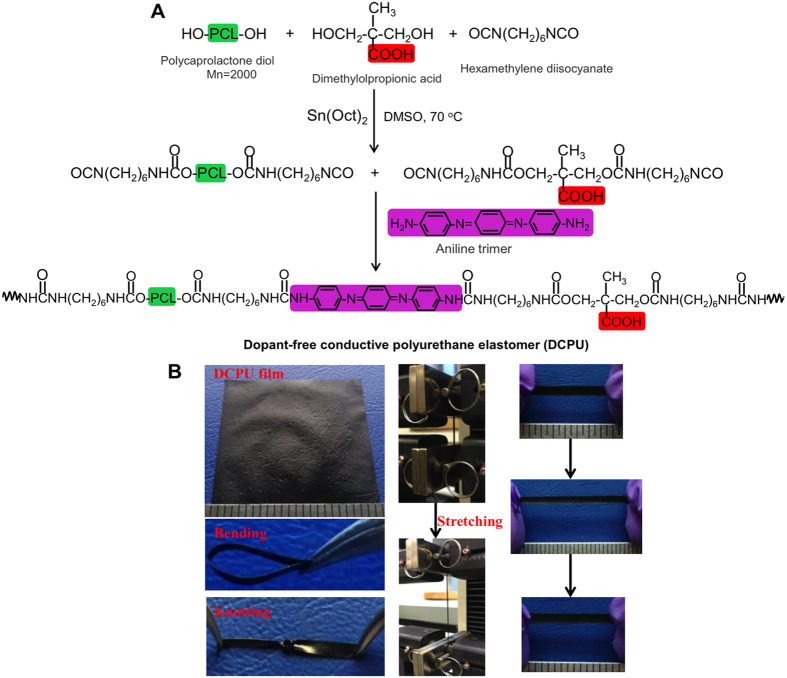
Dopant-free conductive polyurethane elastomer (DCPU) synthesis. (**A**) Synthetic scheme of DCPU. (**B**) Biodegradable DCPU film and its high elasticity presented by bending, knotting, stretching and recoiling.

**Figure 2:**
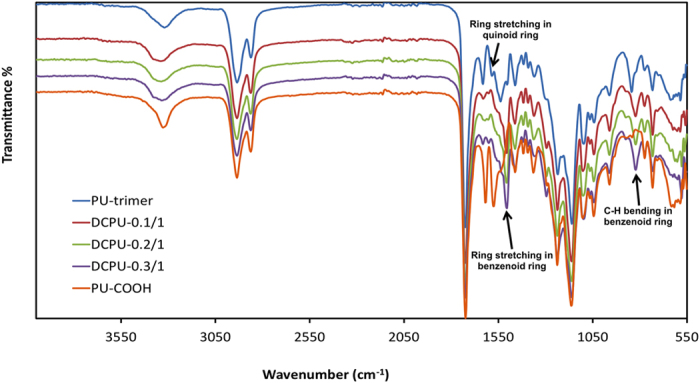
FTIR spectra of DCPU.

DCPU was synthesized from PCL (biodegradable segment), aniline trimer with two amine end groups (conductive segment), and DMPA (dopant molecule) with 1,6-hexamethylene diisocyanate (HDI) using two-step solvent polymerization (Fig. 1A). The PCL:DMPA:HDI:aniline trimer feeding ratios were varied as 0.9:0.1:2:1, 0.8:0.2:2:1, and 0.7:0.3:2:1, which were referred to as DCPU-0.1/1, DCPU-0.2/1, and DCPU-0.3/1 (Table 1). Electroactive DCPU films with high elasticity and flexibility were then obtained (Fig. 1B). Polyurethane without DMPA (PU-trimer) and polyurethane without aniline trimer (PU-COOH) were two control groups. The chemical structure of the DCPU was verified by Fourier transform infrared spectroscopy (FTIR; Fig. 2). The urethane and urea groups were confirmed by specific peaks at 3340 cm^−1^ (N-H stretching of urethane and urea groups), 2940 cm^−1^ and 2860 cm^−1^ (symmetric and asymmetric C-H stretching), 1720 cm^−1^ (C=O stretching of urethane and urea groups)^24^. The specific peaks for aniline trimer were located at 1600 cm^−1^ and 1510 cm^−1^ (ring stretching vibrations of quinoid and benzenoid rings), and 820 cm^−1^ (C-H bending in benzenoid rings)^25^,^26^.

The DCPU polymers had low glass transition temperatures (Tgs) below −60 °C (Table 1), which were attributed to PCL soft segment. The Tg decreased by reducing the PCL amount in DCPU backbone. The melting temperatures (Tms) of DCPU resulted from the semicrystalline PCL segment, and decreased from 29 °C to 24 °C with decreased PCL amount in DCPU backbone. The inherent viscosities of DCPU ranged from 1.20 (DCPU-0.3/1) to 2.32 dL/g (DCPU-0.1/1; Table 1). The water absorption increased with the increasing DMPA amount in DCPU, which was contributed to the hydrophilic carboxyl group on DMPA (Table 1). DCPU-0.1/1 had the lowest water absorption at 8 ± 1%, while DCPU-0.3/1 had the highest water absorption at 15 ± 2%.

**Figure 3:**
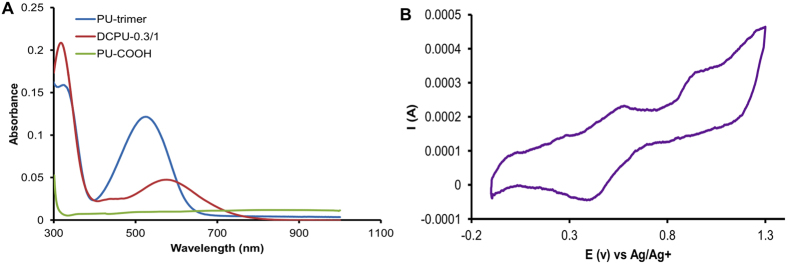
Electroactivity of DCPU. (**A**) UV-vis spectra PU-trimer, DCPU-0.3/1 and PU-COOH in DMF. (**B**) Cyclic voltammogram of DCPU-0.3/1 polymer on Pt electrode in 1.0 M H_2_SO_4_ using Ag/AgCl as reference with scan rate of 50 mV s^−1^.

The UV-vis spectra of PU-trimer, DCPU-0.3/1, and PU-COOH, shown in Fig. 3A show their electroactivities and the effects of the conjugated proton donor (DMPA) on DCPU electroactivity. The PU-trimer had two typical absorption peaks at 526 nm (π_b_-π_q_ transition from the benzene ring to the quinoid ring) and 323 nm (π-π* transition in the benzene ring), which were routinely observed for the emeraldine base form of polyaniline derivatives^27^,^28^. After introducing DMPA into the DCPU backbone, the absorption peak at 526 nm shifted to 578 nm, and a small shoulder band at 438 nm appeared, representing the delocalized polaron peak arising from the polaron-π* transition^2^,^29^. However, the PU-COOH showed no absorption peaks in the wavelength range from 300 nm to 1,000 nm due to the absence of aniline trimer in the polyurethane backbone.

### Electrical and electrochemical properties of DCPUs

The electrical conductivity of DCPU films in dry and wet states is summarized in Table 1. The conductivities of DCPUs in the dry state ranged from 5.5 ± 0.7 × 10^−8^ to 1.2 ± 0.3 × 10^−5^ S cm^−1^. With fixed aniline trimer content, the conductivity of the DCPU rose with an increasing DMPA amount in the polyurethane backbone. The PU-trimer without a dopant possessed very low conductivity at 2.7 ± 0.9 × 10^−10^ S cm^−1^, and the PU-COOH without aniline trimer showed a conductivity value at 5.5 ± 1.2 × 10^−12^ S cm^−1^. The conductivities of DCPUs in the wet state (phosphate buffer solution (PBS) immersion) markedly increased compared to those in the dry state, ranging from 4.4 ± 0.4 × 10^−7^ to 4.7 ± 0.8 × 10^−3^ S cm^−1^. These conductivity values of DCPUs in the wet state showed the same trend as those in the dry state (Table 1). The conductivity of wet PU-COOH was 9.7 ± 0.4 × 10^−8^ S cm^−1^. This increase was attributed to the absorbed PBS in the polymer matrix associated with its bulk hydrophilicity (Table 1). The conductivities of DCPUs were lower than the conductivities of polyaniline (5 S cm^−1^)^1^ and some reported biodegradable conductive materials, such as a polythiophene-based multilayer film (2.7 × 10^−2^ S cm^−1^)^30^ and a polypyrrole-*b*-polycaprolactone (PPy-PCL) copolymer (10–20 S cm^−1^)^14^. However, the conductivities of DCPUs in wet state (from 4.4 ± 0.4 × 10^−7^ to 4.7 ± 0.8 × 10^–3^ S cm^−1^) are also comparable to or higher than some reported conductive biomaterials, which have been applied for neural and myocardial repair^31^,^32^,^33^,^34^,^35^. For example, a blended scaffold of conductive polyurethane containing aniline pentamer and PCL (10^−5^ ± 0.09 S cm^−1^) was capable of improving the adhesion and proliferation of rat neonatal cardiomyocytes^31^,^32^. Polypyrrole-containing nanofibrous scaffolds (1.3 × 10^−5^ to 3.7 × 10^−4^ S cm^−1^) promoted cardiomyocyte attachment, proliferation and interaction as well as cardiac-specific protein expression^33^. Three-dimensional engineered cardiac tissues (ECTs) from single walled carbon nanotubes (SWNTs) and gelatin hydrogels (~10^−3^ S/cm)^34^ enhanced *in vitro* cardiac contraction and the expression of electrochemical associated proteins, and also structurally integrated with the host myocardium and improved heart function in rats^34^. Furthermore, a biodegradable conductive composite made of polypyrrole (2.5% w/w) and chitosan (97.5% w/w) (1.3 ± 0.1 × 10^−3^ S cm^−1^) supported the adhesion, spreading and proliferation of olfactory ensheathing cells with or without electrical stimulation^35^. Thus, it is plausible that the electrical conductivities of DCPU polymers would be sufficient to pass the low micro-current in human bodies and positively affect the cell behaviors such as cell adhesion, proliferation and differentiation^36^,^37^,^38^.

In the cyclic voltammogram of DCPU-0.3/1 (Fig. 3B), the first redox peak at 550 mV represented the reversible redox process from the leucoemeraldine form to the emeraldine form. With higher potentials, the second peak at 930 mV corresponded to the transition from the emeraldine form to the pernigraniline form. However, the PU-trimer and PU-COOH displayed undetectable electrochemical signals due to their poor conductivities (data not shown). The obvious redox peaks corresponding to the transitions of the three oxidation/reduction forms in DCPU-0.3/1 revealed good electroactivity of the DCPU polymer.

### Mechanical properties of DCPU films

**Figure 4:**
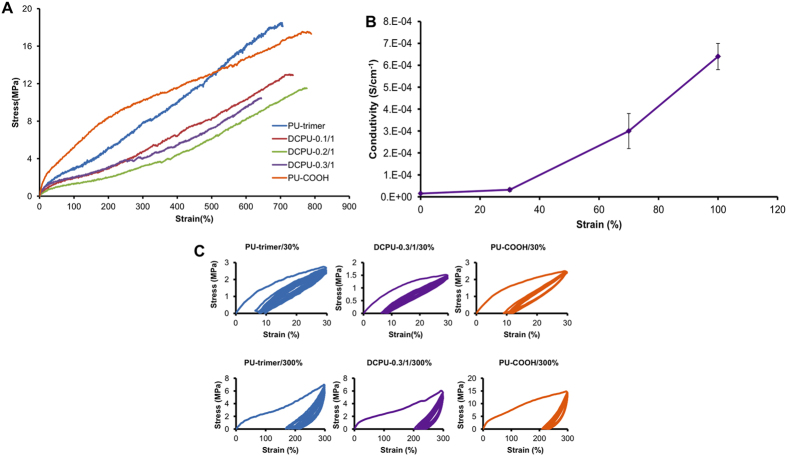
Mechanical properties of DCPU films. (**A**) Stress-strain curves of DCPU films. (**B**) Dependence of electrical conductivity of DCPU-0.3/1 on applied strains varied from 30% to 100%. (**C**) Cyclic stretching of PU-trimer and DCPU-0.3/1 at 30% and 300% deformations.

The DCPU films exhibited robust mechanical properties with softness and high elasticity. The videos and digital images show the attractive mechanical properties of the DCPU polymer, including bending, knotting, stretching, and recoiling (Fig. 1B and Supplementary Video S1). The stress-strain curves of these synthesized polyurethanes showed the typical “S” shape (Fig. 4A), and the tensile strengths and initial moduli of the DCPU films ranged from 9.6 ± 1.2 to 12.6 ± 2.3 MPa and from 3.0 ± 0.6 to 5.2 ± 1.1 MPa, respectively (Table 1). The tensile strengths and initial moduli decreased with increased DMPA content in the DCPU backbone. This might be attributed to the decreased semicrystalline PCL content in the polyurethane backbone, along with reduced PCL crystallinity, which was consistent with the DSC results (Table 1). The breaking strain of the DCPU films ranged from 685 ± 104% to 825 ± 198%, with no significant difference between each group (*p* > 0.05). The instant recovery of all DCPU films was ≥ 99% after three cycles of stretching at 10% strain (Table 1).

The conductivity change of DCPU-0.3/1 was monitored at various uniaxial strains (30%, 70%, and 100% strain) at room temperature (Fig. 4B). There was a slight conductivity increase of the DCPU-0.3/1 film from 1.2 ± 0.3 × 10^−5^ S cm^−1^ (unstretched) to 3.2 ± 0.8 × 10^−5^ S cm^−1^ at a strain of 30%, followed by a sharp rise to 6.4 ± 0.6 × 10^−4^ S cm^−1^ at 100% strain, which was a 43-fold increase compared with that of unstretched DCPU-0.3/1 film. The conductivity increase of the DCPU film by applied strains (from 30% to 100%) was primarily due to the oriented polymer chains along the stretched direction^39^.

To study the resilience of DCPU films, cyclic stretching was performed at a maximum strain of 30% and 300% (Fig. 4C and Supplementary Fig. S1). All the polymers had a large hysteresis loop in the first cycle, followed by smaller hysteresis loops in the next nine cycles. All the samples showed small irreversible deformations (<10%) at a maximum strain of 30%. With the maximum strain of 300%, the irreversible deformations became larger for all polymer samples (~200%). These results verified that DCPUs are robust, elastic, and flexible, which is promising for soft tissue repair and stretchable soft electronics use.

### *In vitro* degradation of DCPU films

**Figure 5:**
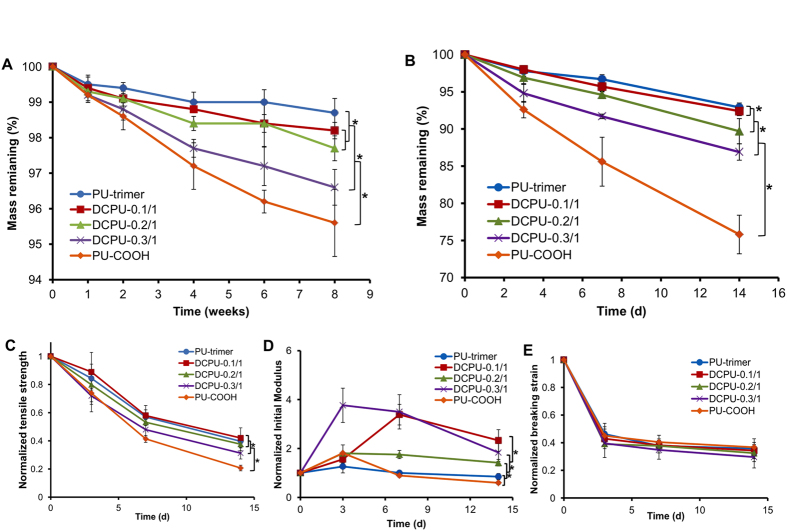
DCPU film degradation. (**A**) Mass remaining for DCPU in PBS at 37 °C. (**B**) Mass remaining for DCPU in 100 U mL^−1^ lipase in PBS solution at 37 °C. The changes of (**C**) tensile strengths, (**D**) initial moduli and (**E**) breaking strains of DCPU films with enzymatic degradation at 37 °C. *Represented significant different groups (*p* < 0.05).

DCPUs could be degraded by hydrolysis and enzymes (Fig. 5A,B). For hydrolytic degradation in PBS (Fig. 5A), DCPU polymers showed low degradation rates in 8 weeks with mass remaining ranging from 96.6 ± 0.5% (DCPU-0.3/1) to 98.2 ± 0.2% (DCPU-0.1/1; *p* < 0.05). The degradation rates of DCPUs increased with an increasing DMPA amount in the DCPU backbone, which resulted from the hydrophilic carboxyl groups in the polyurethane backbone. The higher carboxyl group content allowed more water penetration into the polyurethane film, which then led to faster hydrolysis^40^. Besides, many enzymes exist in the human body that can accelerate the degradation of the polymer *in vivo*^41^. In lipase/PBS solution (Fig. 5B), all polymers degraded faster than in PBS solution. Within 14 days, the polymer degradation behavior showed similar trends as that of DCPUs in PBS. DCPU-0.1/1 had the lowest degradation rate (92.4 ± 0.6% mass remaining), whereas DCPU-0.3/1 had the highest degradation rate (75.8 ± 2.6% mass remaining).

The changes in mechanical property of DCPU films with degradation time were characterized after 3, 7, and 14 days of degradation in lipase/PBS solution (Fig. 5C–E). The tensile strengths of the DCPU films decreased with increasing degradation time. The tensile strength reductions increased with increased hydrophilicity of DCPU polymers. The DCPU-0.3/1 had the highest tensile strength reduction (68.2±4.2% at day 14), and the DCPU-0.1/1 had the lowest tensile strength reduction (57.9±7.1% at day 14). The initial moduli of DCPU films eventually showed a decreasing trend after 14 days of degradation with a temporary increase at the beginning of the degradation period. A possible explanation for this phenomenon could be that at an early stage of enzymatic degradation, soft segment degradation starts earlier and is faster while hard segment degradation may not begin or be slower. This result was comparable to a previous study on the hydrolytic degradation of polyurethanes (PU) synthesized from PCL, 1,4-butanediisocyanate and 1,4-butanediol in PBS at 37 °C over 400 days^42^. It was observed that the Young’s modulus of PU-2300 (containing PCL with molecular weight at 2300 g/mol) increased up to 300 days and then decreased, which was related to the crystallinity changes with time. In addition, the breaking strains of the DCPU films decreased with the enzymatic degradation time without significant difference between each sample (*p* > 0.05).

### Electrical stability of DCPU

**Figure 6:**
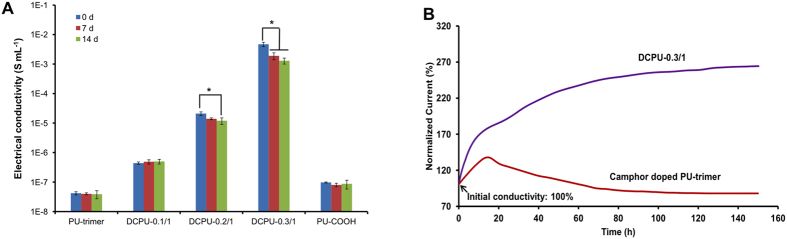
Electrical stability of DCPU. (**A**) Changes in electrical conductivities of DCPU films in lipase/PBS solution within 14 d. (**B**) Relationship between electrical current and incubation time in the electrical stability test of DCPU-0.3/1 film in cell culture medium. Camphor doped PU-trimer film was used as a control.

The electrical conductivity changes of DCPU films with enzymatic degradation up to day 14 are shown in Fig. 6A. The conductivities of DCPU-0.2/1 and DCPU-0.3/1 were slightly reduced from 2.1 ± 0.3 × 10^−5^ S cm^−1^ at day zero to 1.2 ± 0.3 × 10^−5^ S cm^−1^ at day 14, and from 4.7 ± 0.8 × 10^−3^ S cm^−1^ at day zero to 1.3 ± 0.3 × 10^−3^ S cm^−1^ at day 14, respectively (*p* < 0.05). Although the conductivities of DCPU-0.2/1 and DCPU-0.3/1 decreased, the values did not fall below more than an order of magnitude over 14 days of enzymatic degradation. Similar testing was carried out by immersing poly(glycerol-sebacate)/polyaniline composites in PBS solution and recording their conductivity changes every 24 h for a period of 4 days^43^. The conductivities of those composites decreased with time and eventually fell by around an order of magnitude. It must be noted that the enzymatic degradation of polymer in lipase/PBS solution was much faster than the hydrolytic degradation in PBS. Thus, DCPUs may be able to maintain their conductivities for a longer time in a physiological environment, which was further proved by the electrical stability testing of DCPU.

The electrical stability of DCPU-0.3/1 film was conducted in the cell culture medium with a long-term charge of a fixed voltage (Fig. 6B). The detected current changes directly reflected the changes in film conductivity. The current in Fig. 6B was normalized against the initial value at time zero. When the DCPU-0.3/1 film was immersed in the cell culture medium, the current doubled in the first 22 h and then gradually increased. At 150 h, the current reached up to 264% of the initial value. To further demonstrate the good electrical stability of DCPUs, PU-trimer doped with (1S)-(+)−10-camphorsulfonic acid (CSA; the molar ratio of CSA:aniline trimer was set as 1.5:1) was treated under the same conditions (Fig. 6B). After 150 h of charge, the CSA-doped PU-trimer retained only 88% of its initial conductivity. The proton donor (DMPA) was covalently conjugated into the polyurethane backbone, which made the proton donor more difficult to leach out with time or electrical stimulus compared with those free dopants physically mixed in conductive polymers. Furthermore, because of the water absorption ability of DCPU-0.3/1 (15 ± 2%), shown in Table 1, the absorbed cell culture medium with a large amount of electrolytes diffused in polymer matrix made DCPU-0.3/1 possess almost triple conductivity after 150 h of immersion in cell culture medium (264% of initial conductivity)^44^.

The conductive stability of the conductive material is very significant for *in vitro* cell culture and *in vivo* implantation. Conductivity normally decreases with degradation, dopant leaching, and electrical charge (de-doping)^17^,^18^,^43^. For DCPU, the dopant was covalently linked with the polymer, which significantly reduced dopant leaching and de-doping and gave the polymer good conductive stability. Because of the unavoidable wet environment during biomedical applications^45^, DCPUs with good electrical stability have great potential application as electroactive biomaterials.

### Cytotoxicity and cytocompatibility of DCPUs

**Figure 7:**
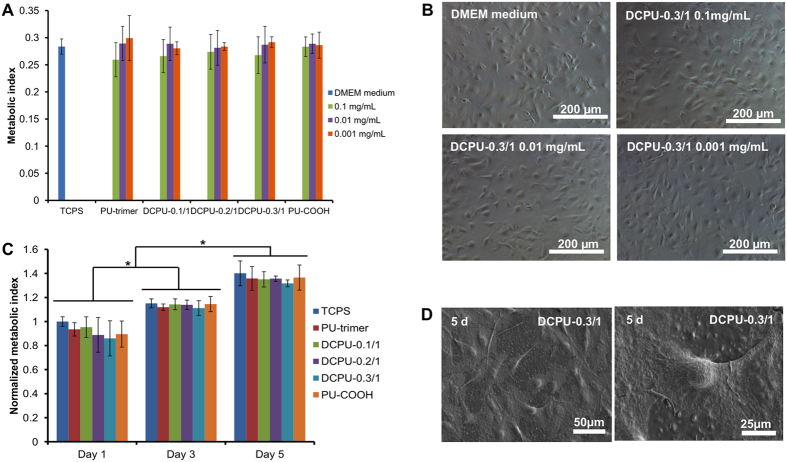
DCPU cytotoxicity and cytocompatibility. (**A**) Metabolic index of mouse 3T3 fibroblasts cultured with DMEM medium mixed with DCPU degradation products at concentrations of 1, 0.1, 0.01 and 0.001 mg mL^−1^. (**B**) Optical microscopy images of mouse 3T3 fibroblasts cultured with DMEM medium mixed with DCPU-0.3/1 degradation products at concentrations of 0.1, 0.01 and 0.001 mg mL^−1^. DMEM medium was used as the control. (**C**) Metabolic index of mouse 3T3 fibroblasts seeded on polyurethane films (TCPS as a control) at days 1, 3 and 5. (**D**) Scanning electron micrographs of mouse 3T3 fibroblasts cultured on the DCPU-0.3/1 film at day 5 (500× and 1000× magnification). *Represented significant different groups (*p* < 0.05).

The cytocompatibility of DCPU films and the cytotoxicity of their degradation products were evaluated using mouse 3T3 fibroblasts (Fig. 7). The cell viability of all DCPU polymers showed no significant difference from that of the Dulbecco’s modified Eagle’s medium (DMEM) control when the concentration of polymer degradation products was ≤0.1 mg mL^−1^ (*p* > 0.05; Fig. 7A). There was no statistical difference between each DCPU polymer group at the same concentration of polymer degradation products (*p* > 0.05). This trend was further visualized by the optical images of 3T3 fibroblasts exposed to different concentrations of DCPU degradation products (Fig. 7B).

Regarding the cytocompatibility of DCPU films (Fig. 7C), the 3T3 fibroblasts proliferated on both DCPU film surfaces and TCPS from 1 to 5 days (*p* < 0.05) with no significant difference between the polymer films and TCPS during 5 days of incubation (*p* > 0.05). The cell morphologies on the DCPU films were visualized by scanning electronic microscope (SEM; Fig. 7D and Supplementary Fig. S2). The cells spread on the polymer films and formed a confluent layer covering the surface of the DCPU films. The magnified SEM image of the single-cell spread on DCPU-0.3/1 film with clear pseudopods is shown in Fig. 3D. The results indicate the good cytocompatibility of DCPU films. We are aware that 3T3 fibroblasts are robust clonal cell line and may have higher tolerance of material toxicity. Future studies should be carried out using specialized and perhaps primary cells to better assess the potential tissue- and cell-specific toxicity of DCPU polymers.

### Fabrication and characterization of porous DCPU scaffold

**Figure 8:**
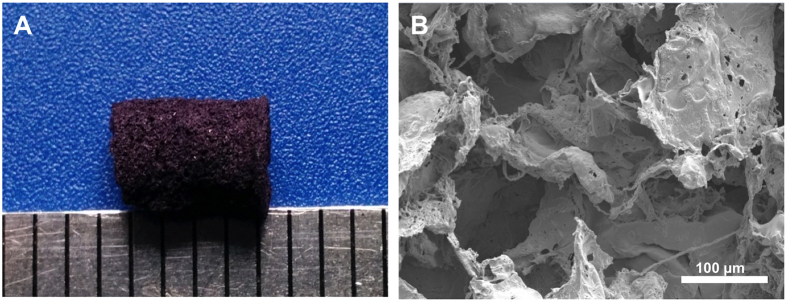
Porous DCPU scaffold. (**A**) Digital image and (**B**) SEM image of porous DCPU-0.3/1 scaffold fabricated by salt leaching.

DCPU can be processed into porous scaffolds using the salt leaching technique (Fig. 8A), which is a convenient way to obtain porous scaffolds with controllable pore size and porosity^31^,^46^. The morphology of DCPU porous scaffolds was observed by SEM (Fig. 8B). The salt particle shaped pores were obvious and interconnected. The pore size and porosity of the DCPU scaffolds were 116 ± 25 μm and 86 ± 4%, respectively. Their elasticity and flexibility is shown in Supplementary Video S1. The poor processability of conventional conductive polymers has limited their biomedical applications due to their poor solubility and flexibility^17^. A conductive polyurethane with aniline pentamer had to be a diffusive additive to blend with PCL to form a film and a porous scaffold because of the poor solubility of this conductive polyurethane, and it still required mixing with a dopant^31^,^32^. However, DCPU as a single-component conductive polymer can be processed into porous conductive scaffolds with flexibility and elasticity without adding extra additives and dopants. This unique property may facilitate the preparation of scaffolds with good material stability and controllability.

### *In vivo* mouse subcutaneous implantation

**Figure 9:**
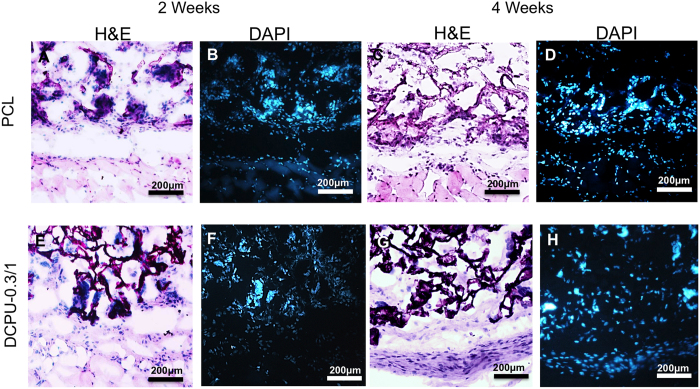
*In vivo* biocompatibility of DCPU porous scaffolds in a mouse subcutaneous model. H&E (**A,C,E,G**) and DAPI (**B,D,F,H**) staining of the tissue surrounding PCL (**A–D**) and DCPU-0.3/1 (**E–H**) porous scaffolds which were implanted for 2 weeks (**A,B,E,F**) and 4 weeks (**C,D,G,H**). PCL scaffolds were used as a positive control.

*In vivo* tissue compatibility of DCPU porous scaffold was evaluated utilizing subcutaneous implantation in mice model for 2 and 4 weeks. Porous scaffold fabricated using PCL, which has excellent tissue biocompatibility and has been used in fabrication of FDA-approved devices, was chosen as a positive control^47^,^48^,^49^,^50^,^51^. The implants and surrounding tissues were stained with H&E and DAPI (nuclei) staining to reflect the extent of tissue compatibility and cell infiltration, respectively (Fig. 9A–H). H&E staining showed that tissue responses to DCPU scaffolds were comparable to those to PCL scaffolds and only small number of inflammatory cells were found at the interface between tissue and both scaffolds at Week 2 (Fig. 9A vs. E) and Week 4 (Fig. 9C vs. 9G). Similarly, we found that both PCL scaffolds and DCPU scaffolds were infiltrated with large number of host cells (stained with DAPI) after implantation for 2 weeks (Fig. 9B vs. 9F) and 4 weeks (Fig. 9D vs. 9H). The *in vivo* results support that DCPU scaffolds have good tissue/cells compatibility while facilitating cell infiltration at the extent similar to PCL scaffolds. Our results support that DCPU possesses excellent cell and tissue compatibility suitable for the fabrication of a variety of tissue engineering scaffolds, medical implants and bioelectronics.

## Conclusion

A biodegradable dopant-free conductive polymer with good elasticity and flexibility was synthesized. Compared to existing biodegradable conductive materials, DCPU is a unicomponent biodegradable elastomer with good electroactivity and electric stability and processability. It also exhibits good cytocompatibility and *in vivo* biocompatibility. The DCPUs may find opportunities to be utilized in tissue repair and regeneration, and other biomedical-related application. This simple and effective methodology can also be utilized to develop serials of novel dopant-free conductive polymers.

## Methods

### Chemical reagents

PCL (average number molecular weight = 2000, Sigma) was dried in a vacuum oven at 60 °C to remove residual water before synthesis. HDI (Sigma) and putrescine (Sigma) were purified by distillation before use. DMPA (Sigma), stannous octoate [Sn(Oct)_2_, Sigma], 4-fluoronitrobenzene (Sigma), *p*-phenylenediamine (Sigma), triethylamine (TEA, Sigma), tin granular (Sigma), ammonium persulfate (Sigma), hydrochloric acid (HCl, Sigma), sodium hydroxide (NaOH, Sigma), CSA (Sigma), anhydrous dimethyl sulfoxide (DMSO, Sigma), acetone (Sigma), 1,1,1,3,3,3-hexafluoroisopropanol (HFIP, Oakwood Product), hexamethyldisilazane (HMDS, Sigma) and lipase from *Thermomyces lanuginosus* (≥100,000 U/g, Sigma) were used as received.

### Synthesis of oxidized aniline trimer with two amine end groups

All chemicals were purchased from Sigma-Aldrich. A round-bottomed flask equipped with a magnetic stirrer and an argon inlet was charged with p-phenylenediamine (1.54 g), 4-fluoronitrobenzene (5.06 g), and triethylamine (2.88 g) in anhydrous dimethyl sulfoxide (DMSO). The reaction lasted 3 days at 125 °C, then was cooled to room temperature, followed by the addition of concentrated HCl, then a red precipitate was formed. The collected red precipitate was subsequently dissolved in concentrated HCl along with granulated tin prior to refluxing for 5 h. A whitish-blue solid was further collected after the addition of concentrated HCl and 5 M NaOH. The solid was then dissolved in ethanol/acetone (1/1, v/v) and 1 M HCl completely, followed by the addition of ammonium persulfate (1.98 g), and stirred in a cold bath for 10 min. The formed blue precipitate was then filtered, washed with an excess amount of distilled water, and dried overnight for the collection of pure oxidized aniline trimer (2.31 g, dark-blue solid). Chemical structure characterization of the oxidized aniline trimer, possessing two NH_2_ end groups, is as follows: ^1^H NMR (500 MHz, DMSO-*d*_6_, δ): 5.43 (s, 4 H), 6.60–6.79 (m, 4 H), 6.89–7.05 (m, 4 H). ^13^C NMR (125 MHz, CDCl_3_, δ): 114.0, 123.0, 124.1, 124.3, 135.2, 136.8, 139.2, 139.3, 147.6, 147.8, 155.1. IR (neat): 3379, 3309, 3206, 1630, 1542, 1318, 1166, 984, 830, 699, 541, 506, 411 cm^−1^. HRMS (ESI) *m/z* calcd for C_18_H_17_N_4_^+^(M + H)^+^ 289.1448; found, 289.1443.

### Synthesis of DCPU

The conductive polyurethanes were synthesized from PCL, DMPA, HDI, and a chain extender aniline trimer. PCL and DMPA were dissolved in DMSO at 70 °C in a three-neck flask under N_2_ protection with stirring, followed by the addition of HDI and 3 drops of catalyst Sn(Oct)_2._ After 3 h of reaction, the prepolymer solution was cooled to room temperature. The aniline trimer/DMSO solution was added dropwise into the pre-polymer solution. The reaction then continued for 18 h at room temperature. The resulting polymer was precipitated in distilled water, washed by ethanol, and then dried in a vacuum oven at 60 °C for 3 days. The molar ratios of PCL:DMPA:HDI:trimer were set as 1:0:2:1, 0.9:0.1:2:1, 0.8:0.2:2:1, and 0.7:0.3:2:1, which were referred to as PU-trimer, DCPU-0.1/1, DCPU-0.2/1, and DCPU-0.3/1, respectively. Polyurethane with a chain extender putrescine (PU-COOH) was used as a control. The molar ratio of PCL:DMPA:HDI:putrescine was 0.7:0.3:2:1. The yields of all final products were above 85%.

### Fabrication of DCPU films

The synthesized DCPU polymers were dissolved in HFIP at a concentration of 2% (wt/v), followed by pouring into a Teflon dish. After the complete evaporation of HFIP, the conductive polymer films were dried in a vacuum oven at 60 °C for 3 days.

### Polymer characterization

FTIR spectra were obtained using a Nicolet 6700 spectrometer (Thermo Scientific, Germany) to verify the chemical structure of DCPU. Thermal properties were characterized by a differential scanning calorimeter (DSC, Shimazu DSC-60) at a scanning rate of 10 °C min^−1^ ranging from −100 to 200 °C with a nitrogen flow. UV-visible spectra of DCPU solutions in DMSO were recorded on a UV-vis spectrometer (PerkinElmer, Lambda 35). For water absorption, the weighted polymer films (W_0_) were incubated in a phosphate buffer solution (PBS, Sigma) at 37 °C. The films were weighted (W_1_) after removing surface water using filter paper. The water absorption was calculated using equation (1):


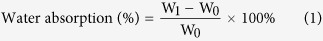


Three parallel samples were tested for each group. The polymer inherent viscosity (IV) associated with molecular weight was measured using an Ubbelohde viscometer^52^. Each sample was dissolved in 15 mL HFIP at a concentration of 0.1 g dL^−1^ and then filtered by a 1.2 μm glass-fiber filter. Each sample was tested five times at room temperature. The IV was calculated using equation (2):


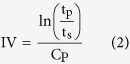


ln (t_p_/t_s_)/C_p,_ where t_p_ is the time for the polymer solution flowing through the capillary; t_s_ is the time for the solvent HFIP flowing through the capillary; and C_p_ is the polymer concentration.

### Electrical conductivity and electrochemical measurements

The electrical conductivity (σ, S cm^−1^) of the DCPU films was measured using the four-point probe technique at both dry and wet states at room temperature^43^,^53^. A direct current (DC) was supplied to pass through the outer probes, and voltage was induced in the inner two probes. The four-point probe was homemade, and the instrument for current supply and voltage measurement was a PARSTAT 2273 potentiostat. The electrical conductivities of the samples were calculated by equation (3):


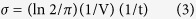


where σ represents the electrical conductivity; I is the current in ampere; V is the voltage in volts; and t is the sample thickness in cm. Four measurements were taken for each group. The electrochemical properties of DCPU were assessed by cyclic voltammetric (CV) analysis using the same potentiostat instrument (PARSTAT 2273) as above^26^. A three-electrode system was involved, consisting of a platinum working sheet electrode coated with the conductive polymer, a platinum-mesh auxiliary electrode, and an Ag/AgCl reference electrode. The CV was recorded at a scan rate of 50 mV s^−1^ in 1 M H_2_SO_4_ solution with scanning potential between −1 and 1.3 V.

### Mechanical testing

The mechanical properties of the samples (2 × 20 mm strips; n = 5) were measured on an MTS Insight Testing System with a 500-N load cell and a crosshead rate of 10 mm min^−1^ according to ASTM D638-03^54^. For conductivity-strain measurements, DCPU films (n = 3) were stretched in the uniaxial direction at room temperature, and then their electrical conductivities at 30%, 70%, and 100% strains were measured using the four-point probe technique, as described above. The instant strain recovery was measured under the same conditions as described above. Two distal ends of the samples were marked, and the samples were stretched to 10% strain, held for 1 min, and released. This stretch cycle was repeated three times. The original length (L_0_) and the length after stretching (L_1_) were measured using a caliper. The instant strain recovery was calculated using equation (4):


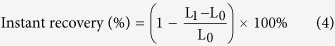


Cyclic stretch testing was conducted by stretching the strips (2 × 20 mm; n = 3) to a maximum strain of 30% or 300%, respectively, and then releasing them back to 0% strain. The stretch cycle was repeated 10 times at a rate of 10 mm min^−1^ 47.

### Polymer degradation

To study the *in vitro* hydrolytic and enzymatic degradation profile of synthesized polymers, the weighted samples (W_0_) were immersed in 10 mL PBS or in 2 mL of PBS containing 100 U mL^−1^ lipase solution at 37 °C^55^. The lipase/PBS solution was changed every 3 days. At a predetermined time point, the samples were rinsed three times with deionized water, dried in a freeze-dryer for 3 days, and then weighed (W_1_). The mass remaining was calculated by equation (5) below. Three parallel samples were used for each group at each time point.


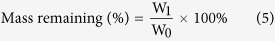


The mechanical properties of the DCPU films (n = 4) after enzymatic degradation were measured as described above.

### Electrical stability of DCPU films

The conductivity changes of the DCPU films (n = 3) were recorded in 100 U mL^−1^ lipase/PBS solution after 7 and 14 days of degradation at 37 °C. At each time point, the degraded DCPU films were taken out and rinsed by PBS to remove the attached enzymes on the film surface. Their conductivities in the wet state were then measured by the four-probe technique, as described above. The electrical stability of the DCPU film was measured in a cell culture medium (Eagle’s medium containing 0.05% sodium azide to prevent bacterial growth) under a constant DC voltage of 100 ± 2 mV provided by a PARSTAT 2273 potentiostat^18^. The incubation lasted for 150 h at 37 °C. The measurement was undertaken in triplicate. PU-trimer doped with CSA (the molar ratio of CSA:aniline trimer was set as 1.5:1) during the film fabrication process as described above was used as a control group. Conductivity changes during enzymatic degradation and electrical stability of the control group (PU-trimer doped with CSA) were measured via the same processes as those of the DCPU films.

### Cytotoxicity of DCPU degradation products

The DCPU polymers (100 mg) were placed in 1 M NaOH solution at 37 °C for 1 week to achieve complete degradation^56^. The degradation solution was neutralized using 10 M HCl solution to pH = 7 and sterilized by a 0.22 μm membrane filter. Mouse 3T3 fibroblasts (ATCC, Manassas, VA) were seeded in 24-well cell culture plates at a density of 1.6 × 10^4^ cells per well in cell culture medium of Dulbecco’s modified Eagle’s medium (DMEM), which was supplemented with 10% fetal bovine serum, 100 U mL^−1^ penicillin, and 100 μg mL^−1^ streptomycin. After 1 day of incubation, the neutralized degradation solution diluted by the DMEM medium at a final concentration of 0.1, 0.01, and 0.001 mg mL^−1^ was then added to each well. The DMEM medium was used as the control group. After 24 h cell culture, the cell viability (n = 4) was measured using a mitochondrial activity assay (MTT, Sigma), and an optical microscope was used to observe cell morphology.

### *In vitro* biocompatibility of polymer films

The polymer films were punched into 6 mm diameter disks using standard biopsy punches (6 mm, Miltex) and sterilized using 70% ethanol solution and UV irradiation for 30 min each, and then they were rinsed by PBS three times. Prior to cell seeding, the sterilized disks were placed in a cell culture medium overnight. Mouse 3T3 fibroblasts were seeded on the sample surface with a seeding density of 3 × 10^3^ per well in 96-well plates. The cell medium was exchanged every 2 days. The MTT assay was used to evaluate the cellular activity (n = 4) at 1, 3, and 5 days. The tissue culture polystyrene (TCPS) was used as a positive control. To qualitatively verify the MTT results and visualize the 3T3 fibroblasts on the films, the cell-seeded films at 1 and 5 days were fixed in 4% paraformaldehyde and dehydrated in graded ethanol solutions (30%, 50%, 70%, 80%, 90%, 95%, and 100%), treated with HMDS, and dried at room temperature. The treated films were observed under SEM (Hitachi S-4800 HRSEM) to visualize the cell morphologies on polymer films.

### Porous scaffold fabrication and characterization

For porous scaffold fabrication, the DCPU-0.3/1 polymer was completely dissolved in HFIP at a concentration of 6% (wt/v). Salt particles (NaCl, Sigma) with sizes ranging from 100 to 150 μm were obtained by American standard sieves. The salt particles (5 g) were uniformly mixed with 1 mL of DCPU/HFIP solution. The DCPU/salt mixture was then placed in a cylinder glass mold and exposed to the air for HFIP evaporation. After complete HFIP evaporation, the scaffold was immersed in DI water for 3 days to remove salt particles. The porous scaffold was eventually obtained after lyophilization for 3 days. The morphology of the porous scaffold was observed under SEM. The scaffold porosity was measured by ethanol displacement^57^. The scaffold sample was immersed in a measurement cylinder containing a known volume of pure ethanol (V_1_). After 5 min, the total volume of ethanol and ethanol-impregnated scaffold was recorded as V_2._ After removing the ethanol-impregnated scaffold from the cylinder, the residual ethanol volume was recorded as V_3_. The scaffold porosity was calculated by equation (6):


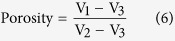


### Mouse subcutaneous implantation model

*In vivo* study was carried out in accordance with National Institutes of Health (NIH) guidelines for animal care and was approved by the Institutional Animal Care and Use Committee of the University of Texas at Arlington. Female Balb/C mice (20–25 grams, purchased from Taconic Farms, Germantown, NY) were utilized for this study. Porous scaffolds made from DCPU-0.3/1 and PCL (a positive control, average Mn = 80,000, Sigma) (4 mm diameter × 2 mm thickness) were implanted subcutaneously on the back of animals. After implantation for 2 and 4 weeks, these mice were sacrificed, and then the implants along with their surrounding tissues were collected and frozen in OCT. For histological analysis of tissue compatibility, 8-μm sections from frozen samples were made using Leica Cryostat (CM1850, Leica 247 Microsystem, Wetzlar, Germany), followed by staining with hematoxylin-eosin (H&E). In addition, 4,6-diamidino-2-phenylindole (DAPI) staining was also performed to assess the extent of cell infiltration in DCPU and PCL porous scaffolds.

### Statistical analysis

All results are presented as mean ± standard deviation. All data were analyzed by one-way ANOVA followed by a post-hoc Tukey-Kramer test. Repeated-measure ANOVA was used for hydrolytic and enzymatic degradation of conductive polymer films using Statistical Analysis System (SAS). *p* < 0.05 was considered a significant difference.

## Additional Information

**How to cite this article**: Xu, C. *et al.* Development of dopant-free conductive bioelastomers. *Sci. Rep.* **6**, 34451; doi: 10.1038/srep34451 (2016).

## Supplementary Material

Supplementary Information

Supplementary Video S1

## Figures and Tables

**Table 1:** Polymer film characterization*.

Samples	Molar ratio of PCL:DMPA:HDI:trimer	Tg (°C)	Tm (°C)	Water absorption (%)	Inherent viscosity (dL/g)	Conductivity (S/cm)		Tensile strength (MPa)	Initial modulus (MPa)	Breaking strain (%)	Instant recovery (%)
						Dry state	Wet state				
PU-trimer	1:0:2:1	−60	32	6 ± 2^a^	2.54	2.7 ± 0.9 × 10^−10^	4.2 ± 0.5 × 10^−8^	17.9 ± 2.0^a^	7.2 ± 0.8^a^	728 ± 88	99 ± 1
DCPU-0.1/1	0.9:0.1:2:1	−61	29	8 ± 1^b^	2.32	5.5 ± 0.7 × 10^−8^	4.4 ± 0.4 × 10^−7^	12.6 ± 2.3^b^	5.2 ± 1.1^b^	695 ± 96	100 ± 2
DCPU-0.2/1	0.8:0.2:2:1	−62	28	9 ± 1^c^	1.37	4.6 ± 0.4 × 10^−7^	2.1 ± 0.3 × 10^−5^	10.9 ± 1.5^b^	3.6 ± 0.4^c^	825 ± 198	99 ± 1
DCPU-0.3/1	0.7:0.3:2:1	−67	24	15 ± 2^d^	1.20	1.2 ± 0.3 × 10^−5^	4.7 ± 0.8 × 10^−3^	9.6 ± 1.2^c^	3.0 ± 0.6^c^	695 ± 104	99 ± 1
PU-COOH	0.7:0.3:2:0^#^	−64	30	19 ± 2^e^	1.23	5.5 ± 1.2 × 10^−12^	9.7 ± 0.4 × 10^−8^	20.3 ± 5.3^a^	16.5 ± 3.1^d^	839 ± 275	99 ± 1

*a, b, c, d, e represent significantly different groups for each characteristic; # Chain extender is putrescine in PU-COOH.
