# Supplementary material for: Development of dopant-free conductive bioelastomers
Source: Sci Rep. 2016 Sep 30;6:34451. doi: 10.1038/srep34451 (PMC5043381; doi:10.1038/srep34451)
Supplement: Supplementary Information [file srep34451-s1.pdf]

Supplementary Information

Development of dopant-free conductive bioelastomers

Cancan Xu[1,2], YiHui Huang[1,2],Gerardo Yepez[3], Zi Wei[4], Fuqiang Liu[4], Alejandro Bugarin[3], Liping Tang[1,2] & Yi Hong[1,2*]

[1]Department of Bioengineering, University of Texas at Arlington, Arlington, TX 76019, USA

[2]Joint Biomedical Engineering Program, University of Texas Southwestern Medical Center, Dallas, TX 75093, USA

[3]Department of Chemistry and Biochemistry, University of Texas at Arlington, Arlington, Texas 76019, USA

[4]Department of Material Science and Engineering, University of Texas at Arlington, Arlington, TX 76019, USA

[*]**Corresponding author:** Yi Hong, yihong@uta.edu, Tel: +1-817-272-0562; Fax: +1-817-272-2251.

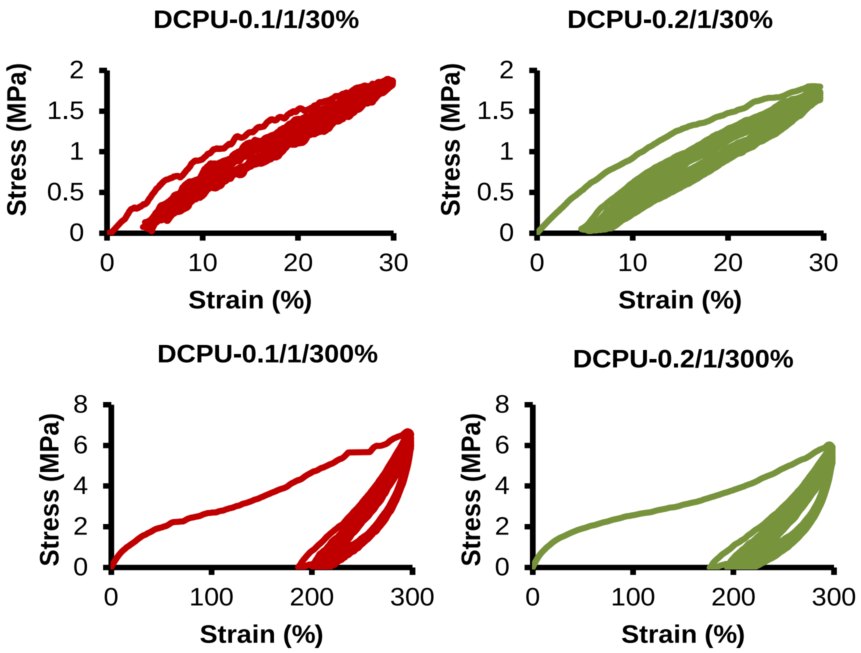

Supplementary Figure S1. Cyclic stretching of DCPU-0.1/1 and DCPU-0.2/1 at 30% and 300% deformations.

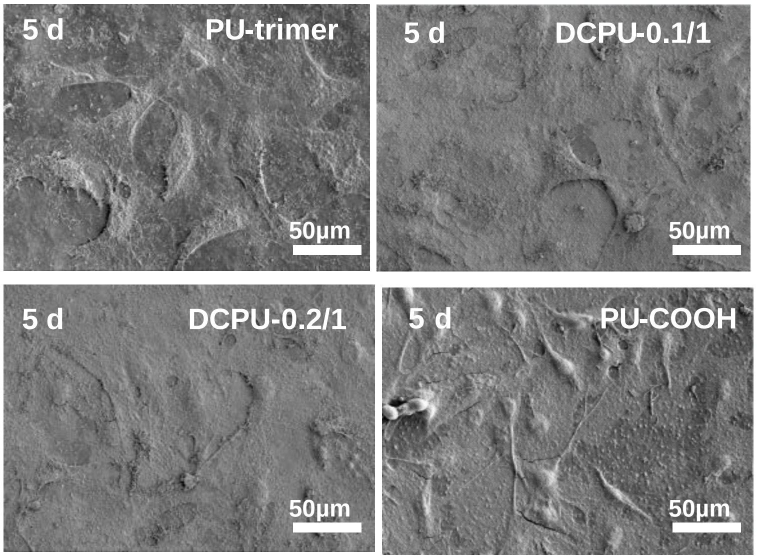

Supplementary Figure S2. Scanning electron micrographs of mouse 3T3 fibroblasts cultured on PU-trimer, DCPU-0.1/1, DCPU-0.2/1 and PU-COOH films at day 5.

Supplementary Video Legend

Supplementary Video S1. DCPU film knotting and stretching, and scaffold compression, which exhibit the softness, stretchability, elasticity and processability of the DCPU.
